# The kinetics of ^18^F-FDG in lung cancer: compartmental models and voxel analysis

**DOI:** 10.1186/s13550-018-0439-8

**Published:** 2018-08-29

**Authors:** Erica Silvestri, Valentina Scolozzi, Gaia Rizzo, Luca Indovina, Marco Castellaro, Maria Vittoria Mattoli, Paolo Graziano, Giuseppe Cardillo, Alessandra Bertoldo, Maria Lucia Calcagni

**Affiliations:** 1 Department of Information Engineering, University of Padova, Via G. Gradenigo 6/B, 35131 Padova, Italy; 2 Department of Diagnostic Imaging, Radiation Oncology and Haematology, Institute of Nuclear Medicine, Fondazione Policlinico Universitario A. Gemelli IRCCS – Università Cattolica del Sacro Cuore, Roma, Italy; 3 Medical Physics Unit, Fondazione Policlinico Universitario A. Gemelli IRCCS, Roma, Italy; 4 Unit of Pathology, Scientific Institute for Research and Health Care “Casa Sollievo della Sofferenza”, San Giovanni Rotondo, Foggia, Italy; 5 Unit of Thoracic Surgery, San Camillo Forlanini Hospital, Rome, Italy

## Abstract

**Background:**

The validation of the most appropriate compartmental model that describes the kinetics of a specific tracer within a specific tissue is mandatory before estimating quantitative parameters, since the behaviour of a tracer can be different among organs and diseases, as well as between primary tumours and metastases. The aims of our study were to assess which compartmental model better describes the kinetics of ^18^F-Fluorodeoxygluxose(^18^F-FDG) in primary lung cancers and in metastatic lymph nodes; to evaluate whether quantitative parameters, estimated using different innovative technologies, are different between lung cancers and lymph nodes; and to evaluate the intra-tumour inhomogeneity.

**Results:**

Twenty-one patients (7 females; 71 ± 9.4 years) with histologically proved lung cancer, prospectively evaluated, underwent ^18^F-FDG PET-CT for staging. Spectral analysis iterative filter (SAIF) method was used to design the most appropriate compartmental model. Among the compartmental models arranged using the number of compartments suggested by SAIF results, the best one was selected according to Akaike information criterion (AIC). Quantitative analysis was performed at the voxel level. *K*_1_, *V*_b_ and *K*_i_ were estimated with three advanced methods: SAIF approach, Patlak analysis and the selected compartmental model. Pearson’s correlation and non-parametric tests were used for statistics. SAIF showed three possible irreversible compartmental models: Tr-1R, Tr-2R and Tr-3R. According to well-known ^18^F-FDG physiology, the structure of the compartmental models was supposed to be catenary. AIC indicated the Sokoloff’s compartmental model (3K) as the best one. Excellent correlation was found between *K*_i_ estimated by Patlak and by SAIF (*R*^2^ = 0.97, *R*^2^ = 0.94, at the global and the voxel level respectively), and between *K*_i_ estimated by 3K and by SAIF (*R*^2^ = 0.98, *R*^2^ = 0.95, at the global and the voxel level respectively). Using the 3K model, the lymph nodes showed higher mean and standard deviation of *V*_b_ than lung cancers (*p* < 0.0014, *p* < 0.0001 respectively) and higher standard deviation of *K*_1_ (*p* < 0.005).

**Conclusions:**

One-tissue reversible plus one-tissue irreversible compartmental model better describes the kinetics of ^18^F-FDG in lung cancers, metastatic lymph nodes and normal lung tissues. Quantitative parameters, estimated at the voxel level applying different advanced approaches, show the inhomogeneity of neoplastic tissues. Differences in metabolic activity and in vascularization, highlighted among all cancers and within each individual cancer, confirm the wide variability in lung cancers and metastatic lymph nodes. These findings support the need of a personalization of therapeutic approaches.

## Background

^18^F-Fluorodeoxygluxose positron emission-computed tomography (^18^F-FDG PET-CT) is one of the most used diagnostic tool in oncology [1]. The quantitative parameters, such as *K*_i_ (the fractional uptake of ^18^F-FDG), *K*_1_ (the rate constant of ^18^F-FDG forward trans-capillary membrane transport) and *V*_b_ (the percentage of blood volume) are not often utilised in clinical practice although they are more reliable than semi-quantitative parameters [2–5] and provide both accurate and exhaustive description of tissue metabolism [6]. Quantitative parameters can be estimated utilising compartmental models solved by non-linear regression methods. In particular, the classical Sokoloff’s compartmental model [7], which was primarily validated to quantify the ^18^F-FDG metabolism in normal brain tissue, in brain tumours and in myocardium [8–10] provides the estimation of four parameters, including *K*_1_ and *V*_b_ plus the derived parameter *K*_i_. Alternatively, *K*_i_ can be obtained by using Patlak graphical analysis [11]. It has been reported that glucose metabolism, represented by the kinetics of ^18^F-FDG, can be different among organs and diseases, as well as between primary tumours and their metastases [12]. Therefore, before estimating quantitative parameters, it is mandatory to design and validate the most appropriate compartmental model for that specific tracer within that specific tissue, rather than to apply “sic et simpliciter” a standard one. Finally, the quantitative analysis can be performed either at a global level, i.e. within the volume of interest, or at a voxel level, applying innovative technologies. Voxel analysis allows identifying the presence of intra-tumour inhomogeneity, which is one of the characteristics of the neoplastic cells. In particular, lung cancer, despite several multimodal therapeutic approaches (http://globocan.iarc.fr/Pages/fact_sheets_cancer.aspx?cancer=lung), still has a poor prognosis mainly due to its intra-tumour inhomogeneity and consequently inhomogeneous response to treatments [13]. Therefore, from a clinical point of view, characterising the intra-tumour inhomogeneity is becoming increasingly more important because it enables us to: (1) better personalise the treatment, such as a tailored radiotherapy planning targeting specific areas within the cancer; (2) better assess the treatment response since it can be inhomogeneous; and (3) better evaluate the prognosis [14]. To our knowledge, a few attempts have been performed in identifying an appropriate compartmental model to describe the kinetics of ^18^F-FDG in lung tissues but in an animal study [15]. And only recently, SAIF has been employed to quantify these kinetics in 5 normal subjects and 11 patients with acute lung injury [16].

The aims of our study were (1) to assess which compartmental model better describes the kinetics of ^18^F-FDG in primary lung cancers and in metastatic lymph nodes; (2) to evaluate whether quantitative parameters can be different between primary lung cancers and metastatic lymph nodes; and (3) to quantitatively investigate the intra-tumour inhomogeneity.

## Methods

### Patients

We prospectively evaluated 21 patients (7 females, 14 males, mean age 71.0 ± 9.4 years) with histologically proved (all but one) primary lung cancer (19 non-small cell lung cancer, 1 non-Hodgkin lymphoma bronchus associated lymphoid tissue) referred from the Unit of Thoracic Surgery of San Camillo Forlanini Hospital of Rome to the PET-CT centre of the Fondazione Policlinico Universitario A. Gemelli IRCCS of Rome.

The characteristics of the patients, the anatomic site of the primary lung cancer and the histopathological data are reported in Table 1. All patients underwent ^18^F-FDG PET-CT for staging, and suspected metastatic lymph nodes with moderate/intense ^18^F-FDG uptake underwent biopsy to confirm their neoplastic nature. Overall, we analysed 23 primary lung cancers (patients no. 8 and no. 15 had two primary lung cancers) and 24 metastatic lymph nodes. Regarding the histological classification, we analysed 9 adenocarcinomas and 11 other histotypes: in one patient (no. 3), two biopsies were both not diagnostic; in patients with two primary lung cancers (no. 8 and no. 15), it has been possible to clearly identify the histotype only in one of them. The ethical committee of Fondazione Policlinico Universitario A. Gemelli IRCCS approved the study, and all patients signed an informed consent form.

**Table 1:** Characteristics of the patients

Patient	Sex	Age	Anatomic site	Histology
1	M	57	Left superior lobe	ADC
2	M	75	Left inferior lobe	NSCLC (favouring adenocarcinoma)
3	F	80	Right superior lobe	Two not diagnostic biopsies
4	M	74	Middle lobe	LCNEC
5	M	55	Left inferior lobe	SqCC
6	M	73	Right inferior lobe	NSCLC (favouring adenocarcinoma)
7	M	50	Right inferior lobe	NHL
8	M	77	Right inferior lobe	SqCC
9	M	80	Left superior lobe	NSCLC (favouring adenocarcinoma)
10	F	73	Left superior lobe	ADC acinar and solid patterns
11	M	73	Right superior lobe	ADC solid pattern
12	M	70	Right superior lobe	NSCLC (favouring adenocarcinoma)
13	M	73	Right Inferior Lobe	ADC acinar and solid patterns
14	F	74	Right Superior Lobe	ADC
15	F	76	Right superior lobe	NSCLC (favouring adenocarcinoma)
16	F	72	Right inferior lobe	ADC acinar and solid patterns
17	M	81	Right superior lobe	ADC
18	F	86	Middle lobe	ADC
19	M	74	Left inferior lobe	NSCLC (favouring adenocarcinoma)
20	M	61	Left superior lobe	ADC
21	F	58	Right superior lobe	NSCLC (favouring adenocarcinoma)

*M* male, *F* female, *ADC* adenocarcinoma, *NSCLC* non-small cell lung carcinoma, *LCNEC* large cell neuroendocrine carcinoma, *SqCC* squamous cell carcinoma, *NHL BALT* non-Hodgkin Lymphoma bronchus associated lymphoid tissue

### ^18^F-FDG PET-CT acquisition protocol and reconstruction data

All patients, fasted for at least 6 h and in normo-glycaemic conditions before they underwent dynamic PET acquisition using an integrated PET-CT scanner (3D Biograph mCT, Siemens Healthcare). An X-ray scout was carried out to precisely define the spatial range of CT acquisition, and a low-dose CT (120 kV, 90 mA) was performed over the thoracic region with a field-of-view of 21 cm. The transaxial CT matrix size was 512 × 512 (1 mm × 1 mm × 3 mm). Patients were intravenously injected with 240 MBq (range 185–333 MBq, according to the body mass index) of ^18^F-FDG, using an infusion pump (Tema Sinergie, model RADInject). 10 ml of ^18^F-FDG were administered at a rate of 4.32 ml/s followed by a 10 ml saline flush. The actual dose delivered to the patient was calculated accounting for the residual activity in the infusion system. PET images were acquired in list mode over the same area defined at a low-dose CT, lasting 60 min. Dynamic PET frames were defined using the following protocol: 24 frames at 5 s, 12 frames at 15 s and 11 frames at 300 s. Each of the 47 frames was reconstructed with the OSEM algorithm, including time-of-flight and UltraHD recovery with 21 subsets and 2 iterations. The transaxial PET matrix size was 256 × 256 (3.18 mm × 3.18 mm × 3 mm). After dynamic acquisition, total-body PET-CT was acquired, and images were reconstructed using the protocol described above. CT images were used for attenuation correction, anatomical localization and fusion with PET images (Syntegra software, Siemens).

### Image processing and quantification

The individual arterial input function was directly derived from the PET images of each patient by manually drawing a region of interest (ROI) in the centre of the descending aorta [17]. The plasma fraction over the blood image-derived activity was calculated assuming a constant partition coefficient of 1.136; derived from Eq. 5 in [18] assuming a lung haematocrit of 40% [19]. ROIs over primary lung cancer and metastatic lymph nodes with ^18^F-FDG uptake were manually drawn on PET-CT images.

^18^F-FDG data were analysed at the voxel level using the semi-quantitative standardized uptake value (SUV) [20] and using three different quantitative methods: Patlak graphical analysis [11], spectral analysis with iterative filter (SAIF) approach as proposed by Veronese and colleagues in [21] and compartmental modelling [22]. As one of the aims of the work was to identify the compartmental model structure that better fits the ^18^F-FDG kinetics in tumours and metastatic lymph nodes, the spectral analysis approach was exploited to identify the number of compartments necessary to describe those kinetics [18]. The component of trapping was explicitly included in the SAIF implementation as from literature it has been know that in lung ^18^F-FDG is irreversibly trapped in the tissues within the first 60 min after injection [23] (i.e. dephosphoryllation is supposed to be negligible over the duration of measurement). In order to configure the structure of the compartmental model, once the number of reversible compartments (“equilibrating components” in [24]) has been provided by SAIF, the compartments were arranged according to physiological insight of the tracer. As previously reported for other outside-brain tissues such as in musculoskeletal tissue [25] and liver [26] (where however the underpinning physiology is different as dephosphoryllation is not negligible), only models with a catenary structure that describe the tracer transport and consumption were considered as physiologically plausible. SAIF results in different scenarios; therefore, for each of them, a compartmental model structure was identified and fitted on the ^18^F-FDG dynamic data using a variational Bayesian approach as proposed by Castellaro and colleagues [27]. The compartmental model that best describes ^18^F-FDG kinetics at the voxel level within both the primary lung cancer and metastatic lymph nodes was finally selected by comparing the fitted models in terms of parsimony using the Akaike information criterion (AIC) [28]. Regarding the model parameters, the inflow rate constant from blood to tissue (*K*_1_, ml/cm^3^/min), the blood volume fraction (*V*_b_, %), and the fractional uptake of ^18^F-FDG (*K*_i_, ml/cm^3^/min) were estimated as main outcome. As in Grecchi et al. [16], *K*_i_ estimated by SAIF approach was chosen as the reference parameter for comparison with *K*_i_ estimated by Patlak analysis and *K*_i_ estimated by the selected compartmental model.

### Statistical analysis

Pearson’s correlation analysis was performed to correlate the *K*_i_ values estimated by Patlak analysis with those estimated by SAIF, as well as the values of *K*_i_ estimated by the compartmental model selected using AIC with those estimated by SAIF. Wilcoxon rank-sum test was employed to compare SUV and quantitative parameters values between primary lung cancers (*n* = 23) and metastatic lymph nodes (*n* = 24). Significance level was set to 0.05. The non-parametric approach was used because of the non-Gaussian distribution of the data, assessed by Komolgorov-Smirnov test. Additionally, to confirm that the statistical results do not depend on outliers, we performed a random cross validation test [29] and bootstrap [30]. Statistical analysis was performed with both in-house and naïve scripts running on Matlab™R2016b (The Mathworks Inc. Natick Massachusetts, USA).

## Results

**Fig. 1:**
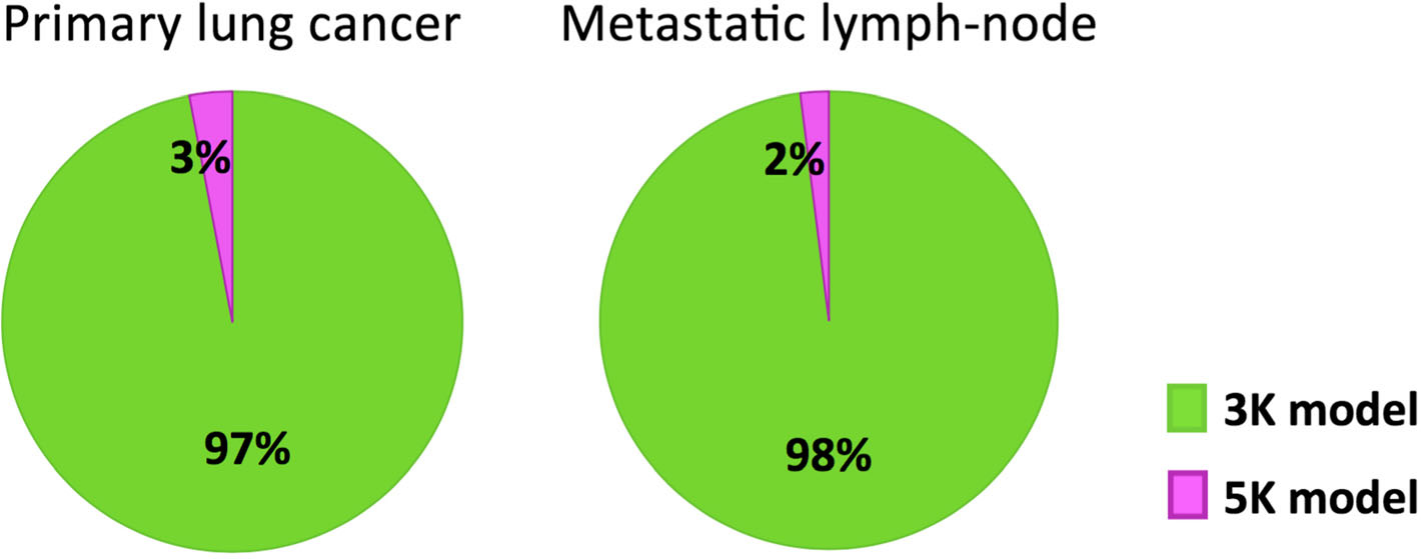
Pie charts of model comparison performed in primary lung cancers and in metastatic lymph nodes. In green, the percentage of voxels for which Akaike information criterion indicates the 3K compartmental model as the optimal model to fit ^18^F-FDG data. In violet, the percentage of voxels for which the 5K compartmental model results the optimal model

In primary lung cancers, in metastatic lymph nodes and in normal lung tissues, SAIF approach showed one trapping component and up to three spectral lines corresponding to just as many reversible compartments. The model order related to these results is reported in what follows as Tr-1R, Tr-2R and Tr-3R, where Tr and R stand respectively for trapping and reversible compartment. Table 2 reports the percentages of number of compartments identified at the voxel level within primary lung cancers, metastatic lymph nodes and in normal lung parenchyma of all patients. Among the possible compartmental models provided by SAIF, the Tr-3R occurred in less than 3% of voxels; hence, it was decided to not consider it for further analysis as scarcely plausible. Starting from the SAIF results (i.e. trapping plus one or two reversible compartments), two catenary models were defined, namely the 3K and the 5K model. The 3K model includes one reversible compartment followed by one irreversible compartment: the first compartment represents the exchanges from plasma pool to interstitial-intracellular space and back, while the irreversible compartment describes the phosphorylation process. The 5K model includes two reversible compartments followed by one irreversible compartment; in this second model, the first and second compartments respectively represent the transport between plasma pool and the extracellular space and between the latter and the cell; as for 3K, phosphorylation is described by the irreversible compartment. After estimating 3K and 5K model with a variational Bayesian approach, the AIC indicated that in almost all voxels of the primary lung cancers and metastatic lymph nodes, 3K represented the best compromise between model fit and model complexity (Fig. 1).

**Table 2:** Number of compartments that SAIF approach returned to describe the kinetics of ^18^F-FDG. Average and standard deviation among patients of the percentages of voxels resulting from SAIF in one, two or three reversible compartments (plus trapping) are reported

	SAIF		
	One-tissue reversible compartment (Tr-1R)	Two-tissue reversible compartment (Tr-2R)	Three-tissue reversible compartment (Tr-3R)
Primary lung cancers (mean ± SD)	56% ± 13%	41% ± 12%	3% ± 3%
Metastatic lymph nodes (mean ± SD)	59% ± 11%	39% ± 10%	2% ± 2%
Normal lung tissues (mean ± SD)	57% ± 10%	40% ± 9%	3% ± 2%

*SAIF* Spectral Analysis with Iterative Filter, *SD* standard deviation

In primary lung cancers and in metastatic lymph nodes, the correlation between *K*_i_ values estimated by Patlak analysis with those estimated by SAIF, as well as between *K*_i_ values estimated by 3K model with those estimated by SAIF was high both at the global level (*R*^2^ = 0.97 and *R*^2^ = 0.98, respectively) and at the voxel level (*R*^2^ = 0.94, *R*^2^ = 0.95, respectively).

**Fig. 2:**
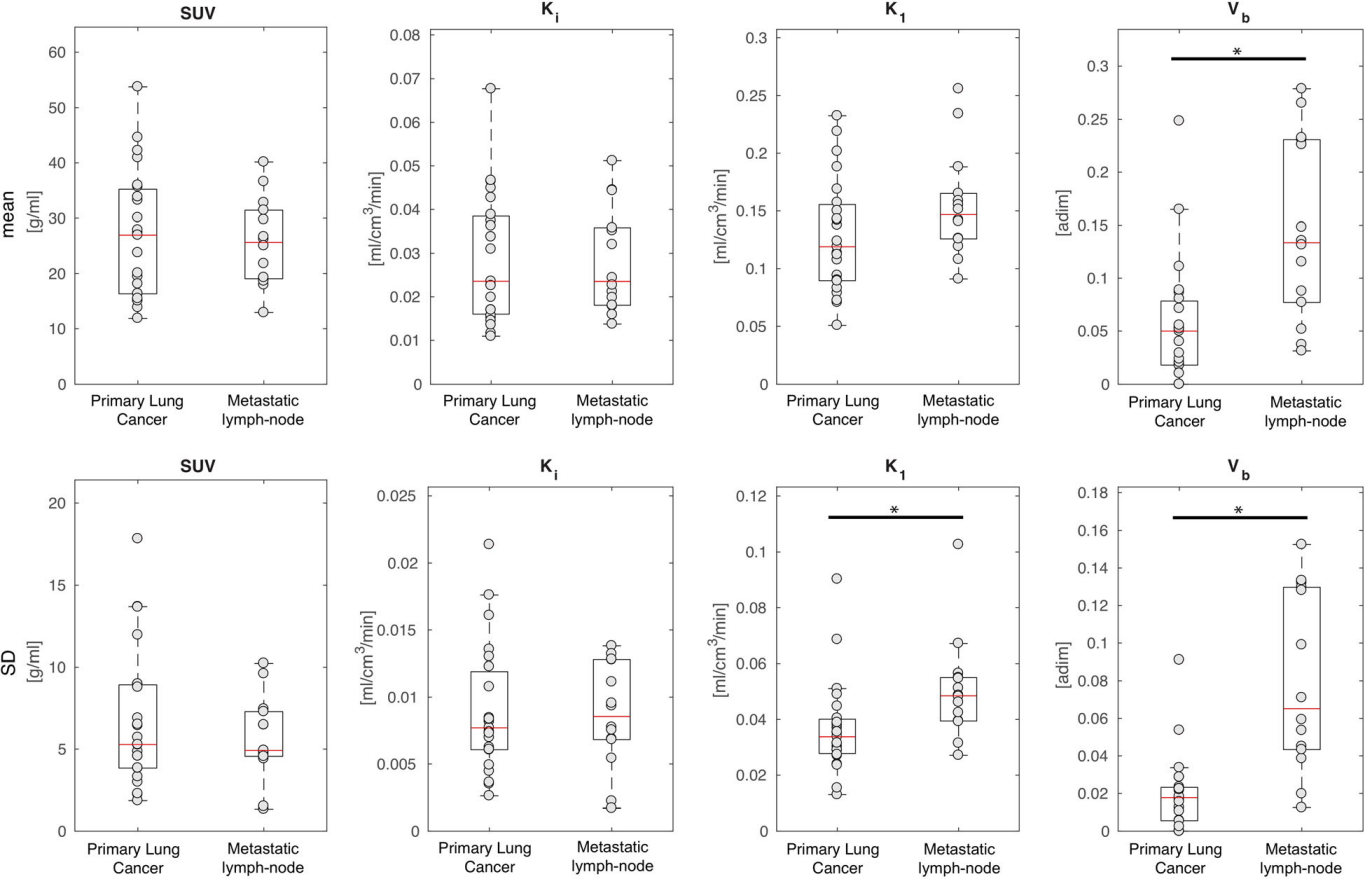
Comparison of quantitative parameters estimated by 3K model in primary lung cancers and metastatic lymph nodes. Box plot of mean values (top row) and standard deviation (SD, bottom row) of *K*_i_, *K*_1_ and *V*_b_, respectively, in primary lung cancers (*n* = 23) and metastatic lymph nodes (*n* = 24). The individual values making up the box plot are shown as grey points. In the box plots, the red line indicates the median value and the edges of the box plot are the 25th and 75th percentiles, whereas the star (*) indicates statistically significant differences between the two groups (*p* < 0.05, Wilcoxon rank-sum test)

Figure 2 shows the mean and standard deviation values of *K*_i_ (i.e. the ^18^F-FDG net uptake of the tracer), *K*_1_ (i.e. the ^18^F-FDG transport rate from plasma to tissue, which is tightly related to the blood flow) and *V*_b_ (i.e. the blood volume fraction), estimated by 3K model, in both primary lung cancers (*n* = 23) and in metastatic lymph nodes (*n* = 24). Metastatic lymph nodes showed significant higher mean and standard deviation values of *V*_b_ when compared with lung cancers (*p* < 0.0014, *p* < 0.0001 respectively), as well as higher standard deviation values of *K*_1_ (*p* < 0.005).

For completeness, we have also tested for differences between primary lung cancers and metastatic lymph nodes in *k*_2_ (i.e. ^18^F-FDG transport from the interstitial-intracellular space to the blood) and *k*_3_ (i.e. phosphoryllation) parameters (results not reported in Fig. 2 for the 281 sake of clarity), as well as in SUV, however, no statistically significant difference was found.

**Fig. 3:**
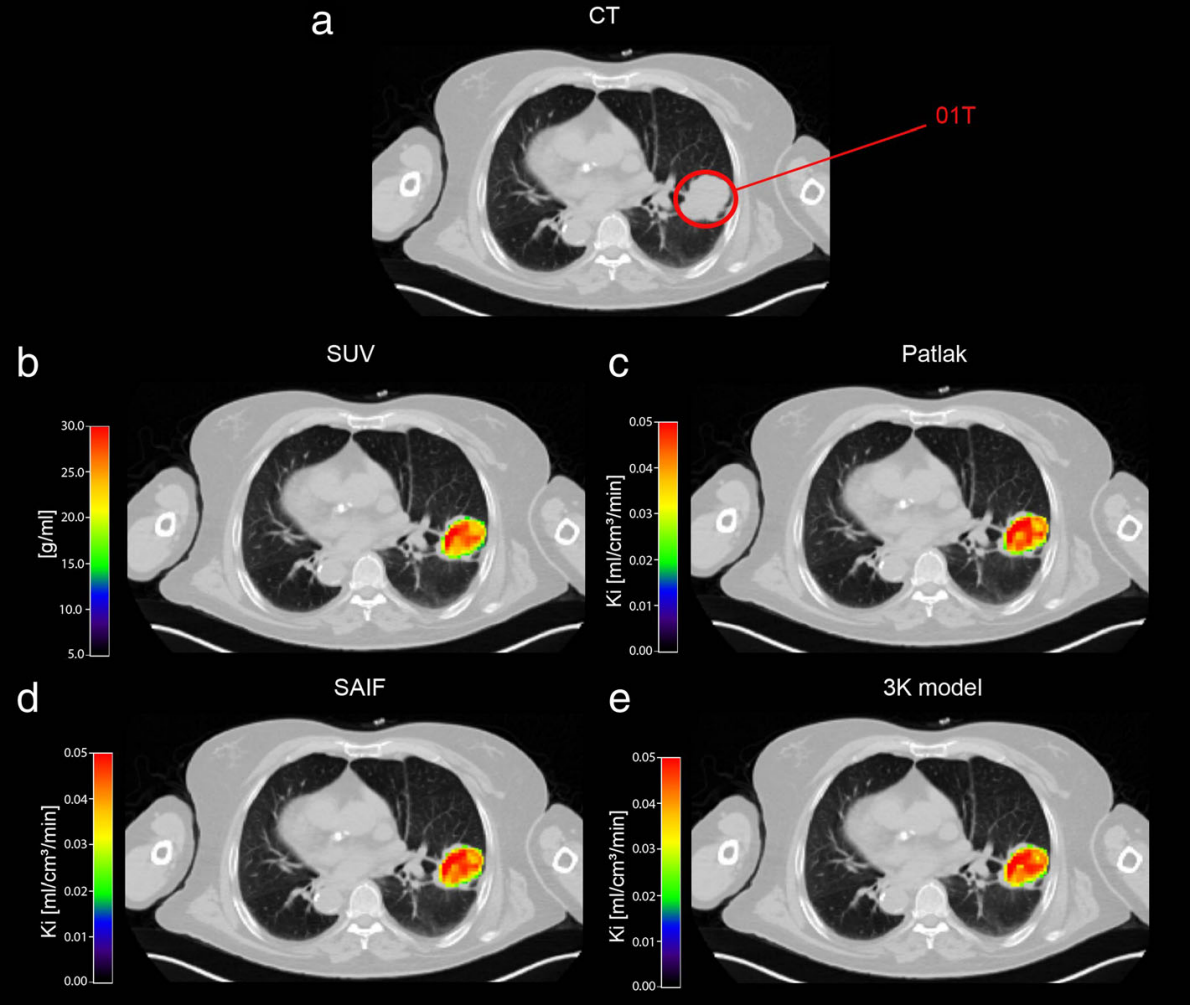
Parametric maps of SUV and *K*_i_ in a representative patient. Transaxial thoracic CT image: the primary lung cancer is highlighted by red circle (**a**). Map of SUV within primary lung cancer over-imposed on transaxial thoracic CT image (**b**). Voxel-wise maps of *K*_i_ obtained with Patlak, SAIF approach and 3K model, respectively, in primary lung cancer over-imposed on transaxial thoracic CT image (**c**, **d** and **e**) *CT*, computed tomography; *SUV*, standardized uptake value; *K*_i_, the fractional uptake of ^18^F-FDG; *SAIF*, Spectral Analysis with Iterative Filter.

**Fig. 4:**
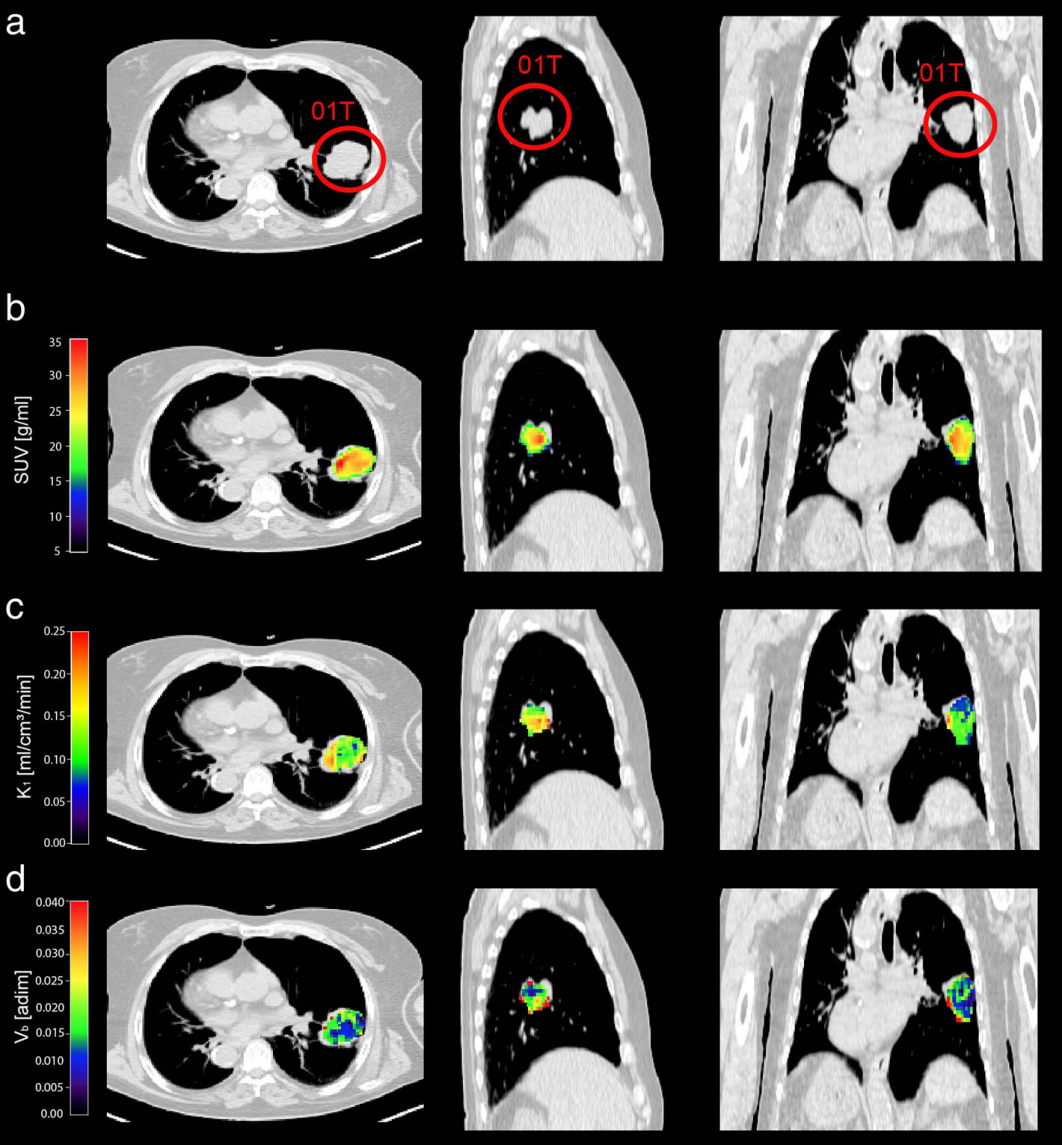
Parametric maps of SUV, K_1_ and V_b_ in a representative patient. Transaxial, lateral and frontal views of thoracic CT image: the primary lung cancer is highlighted by red circle (**a**). Map of SUV (**b**) within primary lung cancer over-imposed on thoracic CT image (**b**). Voxel-wise parametric maps of *K*_1_ and *V*_b_ obtained with 3K model in primary lung cancer over-imposed on thoracic CT image (**c** and **d**). *CT*, computed tomography; *SUV*, standardized uptake value; *K*_1_, ^18^F-FDG transport rate from plasma to tissue; *V*_b_, blood volume fraction

Figures 3 and 4 report respectively, in a representative patient, the map of SUV and the parametric maps of *K*_i_ values obtained with Patlak analysis, SAIF approach,and 3K model, and the parametric maps of *K*_1_ and *V*_b_ as well as the spatial distribution of SUV. The heterogeneity of ^18^F-FDG-uptake distribution, influx rate constant and blood volume fraction within voxels in primary lung cancer is evident.

## Discussion

This is the first paper that identifies, in oncological patients, which compartmental model better describes the kinetics of ^18^F-FDG in both primary lung cancers and in their metastatic lymph nodes. The application of a single “standard compartmental model” to different organs and/or to different diseases is not recommended. Therefore, before estimating quantitative parameters, it is important to understand the behaviour of the tracer in that organ/disease and to individuate the most appropriate compartmental model to describe it. A very few authors followed this approach in animals [15].

From our data, SAIF approach performed at the voxel level showed that in primary lung cancers, metastatic lymph nodes and normal lung tissues, the kinetics of ^18^F-FDG could be represented by compartmental models with three different model orders: one-tissue reversible plus one-tissue irreversible (Tr-1R), two-tissue reversible plus one-tissue irreversible (Tr-2R) and three-tissue reversible plus one-tissue irreversible (Tr-3R). Percentages obtained in normal lung tissues reported in Table 2 are in line with the findings of Grecchi and colleagues [16], whereas the very low percentages obtained for the Tr-3R in the tumour and metastases tissues led us to consider this configuration as scarcely plausible and to exclude it from the further compartmental modelling and model selection. According to the SAIF results and the physiological knowledge on ^18^F-FDG transport and consumption [25, 26], two catenary model structures were tested: 3K and 5K. Between 3K and 5K compartmental models, AIC selected the 3K model as the best to describe the kinetics of ^18^F-FDG within tumours and metastatic lymph nodes. Differences between the percentages obtained with SAIF and AIC in terms of model complexity (i.e. number of spectral lines in the first case and number of model parameters in the second) are somehow expected, as the mathematical framework that lies at the base of those two methods is very different. In particular, the spectral analysis approach relies on a linear decomposition of the dynamic data that employs an overcomplete basis of the signals space [31] which makes the decomposition not unique and could lead to an overestimation of the number of compartments necessary to describe the kinetics. Whereas, AIC is employed for the model selection in a compartmental modelling framework, where the structure of the two compared models is fixed (i.e. it has not to be estimated), and the selection is based on a compromise between the model complexity and the accuracy of the model prediction. Notably, the 3K is the same model validated to quantify the ^18^F-FDG-glucose metabolism in normal brain tissue, in brain tumours and in myocardium [8–10] and has the same complexity observed in normal and inflamed lung tissue [16]. Therefore, these findings confirm that 3K compartmental model can be applied to analyse the kinetics of ^18^F-FDG in normal lung tissue but also in lung cancer.

Regarding the quantitative parameters, excellent correlation was found between *K*_i_ estimated by Patlak and by SAIF (*R*^2^ = 0.97, *R*^2^ = 0.94, at the global and the voxel level respectively) and between *K*_i_ estimated by 3K and by SAIF (*R*^2^ = 0.98, *R*^2^ = 0.95, at the global and the voxel level respectively). Therefore, the three mathematical methods are equally good at estimating *K*_i_. From a clinical point of view, we advise the use of Patlak graphical analysis because of its robustness and simplicity, and when it is sufficient to have only the estimation of *K*_i_. More advanced methods, such as compartmental modelling or SAIF, are recommended when it is important to provide a full characterisation of the tracer kinetics [6].

As clearly depicted in Fig. 4, the quantitative parameters, when obtained at the voxel level with a compartmental modelling approach, allow to better point out the intra-tumoural inhomogeneity if compared with SUV maps. It is well known that cancers are characterised by areas of higher or lower or absence functionality when compared with healthy tissues. This is due to several factors, such as cellular proliferative activity, hypoxia, necrosis, angiogenesis, gene expression, etc. [32–36]. Quantitative parameters identified at a voxel level have a potential role in several phases of the lung tumour care. In particular, the diagnostic process could benefit of a better tissues characterisation, as well as the treatment planning where the delivered radiation dose need to be modulated within tumour according to its pathophysiology. Moreover, the ^18^F-FDG absolute quantification at the voxel level enables to assess the spatially heterogeneity of tissues which is relevant to evaluate the treatments response and to accurately estimate the patient’s prognosis [37–39].

From our data, we found that the ^18^F-FDG uptake rate, the ^18^F-FDG consumption and the blood volume fraction are variable among all primary lung cancers, as well as among all metastatic lymph nodes as expressed by relatively wide range of *K*_1_, *K*_i_, and *V*_b_ mean values depicted in Fig. 2 (inter-cancer variability). In addition, within each individual cancer (primary or metastatic lymph node), we found inhomogeneity of functional activity with areas of faster and slower rate of ^18^F-FDG uptake and metabolic activity, and areas heterogeneously vascularized as expressed by relatively wide range of standard deviation of all parameters evaluated (intra-cancer variability). Differences in functional activity and vascularization are expected because it is known that the behaviour of neoplastic cells is variable and depends on histotype, histological grading (inter-cancer variability), as well as on up-regulation of glucose transporters and hexokinase enzymes, aggressiveness, hypoxia, etc. (intra-cancer variability) [40–43]. Finally, we observed that the inhomogeneity is more evident in metastatic lymph nodes compared with primary lung cancers, as expressed by significant higher standard deviation values of *K*_1_ and *V*_b_ (*p* < 0.05 by Wilcoxon rank-sum test). This finding suggests that metastases tend to be less differentiated with a more chaotic behaviour compared with primary cancers [14, 44, 45]. The metastatic lymph nodes are characterised by higher blood volume and higher ^18^F-FDG-uptake rate (even if, this latter, not significant) when compared with primary lung cancers. Furthermore, these findings might be attributed to higher up-regulation of glucose transporters [46], poor differentiation, higher angiogenesis, faster growing and higher aggressiveness of the metastases compared with primary cancers [14, 44, 45]. In clinical practice, it is difficult to evaluate the intra-cancer inhomogeneity even with biopsy, which is considered the gold standard, because when the result is positive for malignancy it might be representative only of a small part of the sample. Moreover, in some cases, the result of biopsy may be not diagnostic, due to insufficient materials, or negative for malignancy because the neoplastic cells are not included in the sample. In addition, it is almost impossible to perform biopsy of all metastatic tissues to evaluate intra-cancer inhomogeneity. The voxel-analysis has the potential to provide a more extensive map of the intra-cancer inhomogeneity of all neoplastic sites, or at least of those included in the field of view of the tomograph.

Finally, regarding SUV, we did not find any significant difference, also at voxel level, between primary lung cancers and metastatic lymph nodes. This finding further confirms that quantitative parameters, obtained from a full dynamic study, highlight more detailed aspects of the tracer kinetics compared to SUV, obtained with a simple static acquisition.

Limitations of the study were as follows: the small sample size due to the difficulty to have surgical specimens of all neoplastic lesions in patients with advanced disease; and the possibility to evaluate only the mediastinal lymph nodes included in the field of view of the tomograph.

Of note, our data were not corrected for motion presence. Motion can have an impact on the assessment of model parameters. In fact, normal respiratory motion, involuntary patient motion and cardiac cyclic movement can introduce partial volume effect in the ROIs and, consequently, reduce the precision and accuracy of the estimates.

It is known that the smaller the ROI size the higher the impact on the estimates will be [47]. In our study, the smaller ROIs included in the analysis have a minimum size of 13.5 mm (patient no. 1). Thus, we do not expect a misinterpretation of the results due to this issue.

It is also known that motion reduces and regularises the PET intensity [47]. This decreases the power of our quantitative analysis in detecting tissues heterogeneity. In other words, it is expected that tissue heterogeneity is underestimated in our study.

However, these limitations are not strictly related to the dynamic acquisition since, these factors (i.e. normal respiratory motion, involuntary patient motion and cardiac cyclic) have an impact even on the static SUV images [48].

## Conclusions

In primary lung cancers, in metastatic lymph nodes and in normal lung tissue, the kinetics of ^18^F-FDG can be described by one-tissue reversible plus one-tissue irreversible compartmental model (3K). The quantitative parameters, especially when estimated at the voxel level using advanced approaches, show the finest differences in the kinetics of ^18^F-FDG reflecting the inhomogeneity of the neoplastic tissues. The differences in metabolic activity and in vascularisation, highlighted among all cancers and within each individual cancer confirm the wide inter- and intra-cancer variability of primary lung cancer and metastatic lymph nodes. Further studies are needed in larger and more selected oncological samples to confirm that quantitative parameters at the voxel level are useful especially to better individualise the treatment and to evaluate both the treatment response and the prognosis. In addition, to validate the method, it would be interesting to correlate the quantitative parameters representing vascularization and metabolism with immunohistochemical analysis.
